# Supplementary material for: Caenorhabditis elegans LET-413 Scribble is essential in the epidermis for growth, viability, and directional outgrowth of epithelial seam cells
Source: PLoS Genet. 2021 Oct 21;17(10):e1009856. doi: 10.1371/journal.pgen.1009856 (PMC8570498; doi:10.1371/journal.pgen.1009856)
Supplement: S1 Fig — Expression of GFP::AID::LET-413 in the pharynx (left), reproductive system (middle), and excretory canal (right) of L4 stage GFP::AID::let-413 animals (strain BOX466). Sp: spermatheca, Ut: Uterus, Vul: Vulva. Related to Fig 1. (PDF) [file pgen.1009856.s001.pdf]

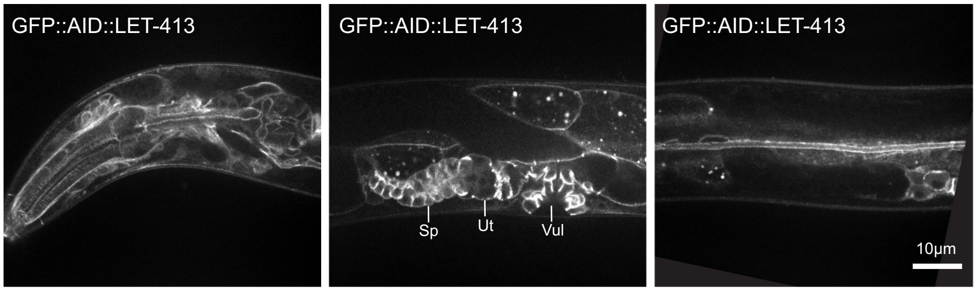

S1 Fig. Larval expression of LET-413. Expression of GFP::AID::LET-413 in the pharynx (left), reproductive system (middle), and excretory canal (right) of L4 stage *GFP::AID::let-413* animals (strain BOX466). Sp: spermatheca, Ut: Uterus, Vul: Vulva. Related to Fig 1.
