# Supplementary material for: Caenorhabditis elegans LET-413 Scribble is essential in the epidermis for growth, viability, and directional outgrowth of epithelial seam cells
Source: PLoS Genet. 2021 Oct 21;17(10):e1009856. doi: 10.1371/journal.pgen.1009856 (PMC8570498; doi:10.1371/journal.pgen.1009856)
Supplement: S2 Fig — Time series of L2 and L3 seam cells divisions and subsequent extension in LET-413-depleted (+auxin) or control animals (-auxin) (strain BOX582). Seam-specific GFP::H2B and GFP::PH mark DNA and cell membrane, respectively. Times indicate hours post hatching. For the L2 division pattern (blue), auxin was added from 8h after hatching, and for the L3 division pattern (red) from 19h after hatching. Related to Fig 3. (PDF) [file pgen.1009856.s002.pdf]

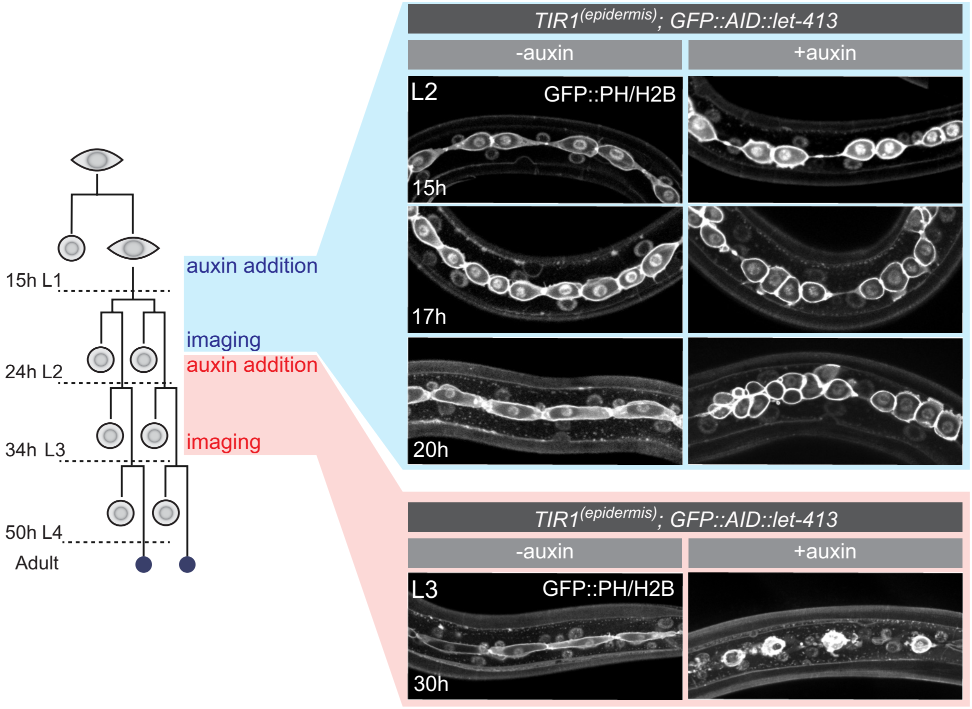

S2 Fig. LET-413 is required for seam cell outgrowth throughout development. Time series of L2 and L3 seam cells divisions and subsequent extension in LET-413-depleted (+auxin) or control animals (-auxin) (strain BOX582). Seam-specific GFP::H2B and GFP::PH mark DNA and cell membrane, respectively. Times indicate hours post hatching. For the L2 division pattern (blue), auxin was added from 8h after hatching, and for the L3 division pattern (red) from 19h after hatching. Related to Fig 3.
