# Supplementary material for: Caenorhabditis elegans LET-413 Scribble is essential in the epidermis for growth, viability, and directional outgrowth of epithelial seam cells
Source: PLoS Genet. 2021 Oct 21;17(10):e1009856. doi: 10.1371/journal.pgen.1009856 (PMC8570498; doi:10.1371/journal.pgen.1009856)
Supplement: S3 Fig — (A) Q cell descendant QRa and QRb during anterior migration (4–5 h post hatching), marked with epidermal-specific mCherry::H2B and mCherry::PH (strain BOX531). No expression of GFP::AID::LET-413 is detected. (B) Migration and division of Q cell descendants in LET-413-depleted (+auxin) or control animals (-auxin) at 2–3 h and 4–5 h post hatching (strain BOX582). Related to Fig 3. (PDF) [file pgen.1009856.s003.pdf]

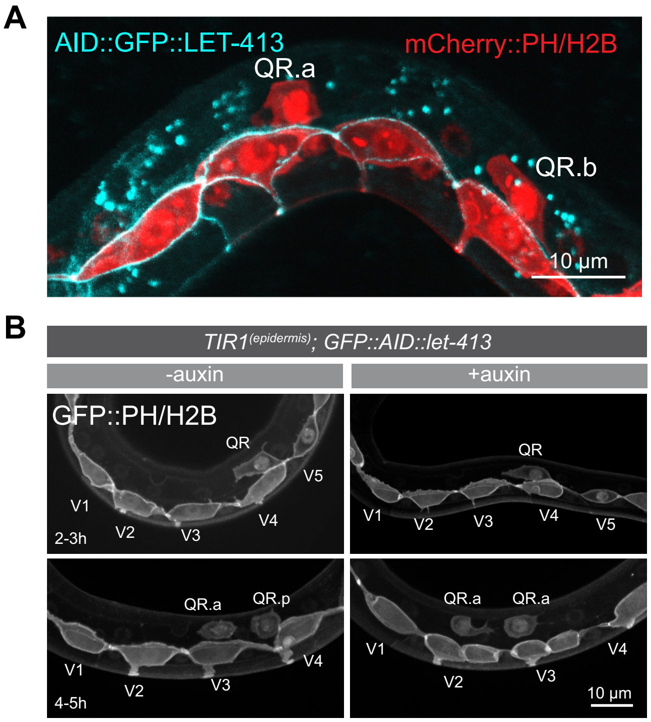

S3 Fig. Degradation of LET-413 in the epidermis does not affect Q cell migration. (**A**) Q cell descendant QRa and QRb during anterior migration (4–5 h post hatching), marked with epidermal-specific mCherry::H2B and mCherry::PH (strain BOX531). No expression of GFP::AID::LET-413 is detected. (**B**) Migration and division of Q cell descendants in LET-413-depleted (+auxin) or control animals (-auxin) at 2–3 h and 4–5 h post hatching (strain BOX582). Related to Fig 3.
