# Supplementary material for: Caenorhabditis elegans LET-413 Scribble is essential in the epidermis for growth, viability, and directional outgrowth of epithelial seam cells
Source: PLoS Genet. 2021 Oct 21;17(10):e1009856. doi: 10.1371/journal.pgen.1009856 (PMC8570498; doi:10.1371/journal.pgen.1009856)
Supplement: S4 Fig — Distribution of DLG-1::mCherry in the epidermis of Pwrt-2::TIR1::BFP; GFP::AID::let-413; dlg-1::mCherry animals without (-auxin) and in the presence of auxin (+auxin) at 5 h and 7 h post hatching (strain BOX527). Boxed region in top overview panels is shown enlarged below. Related to Fig 5. Note that 7 h timepoint is also shown in Fig 5E, and replicated here for ease of comparison between time points. (PDF) [file pgen.1009856.s004.pdf]

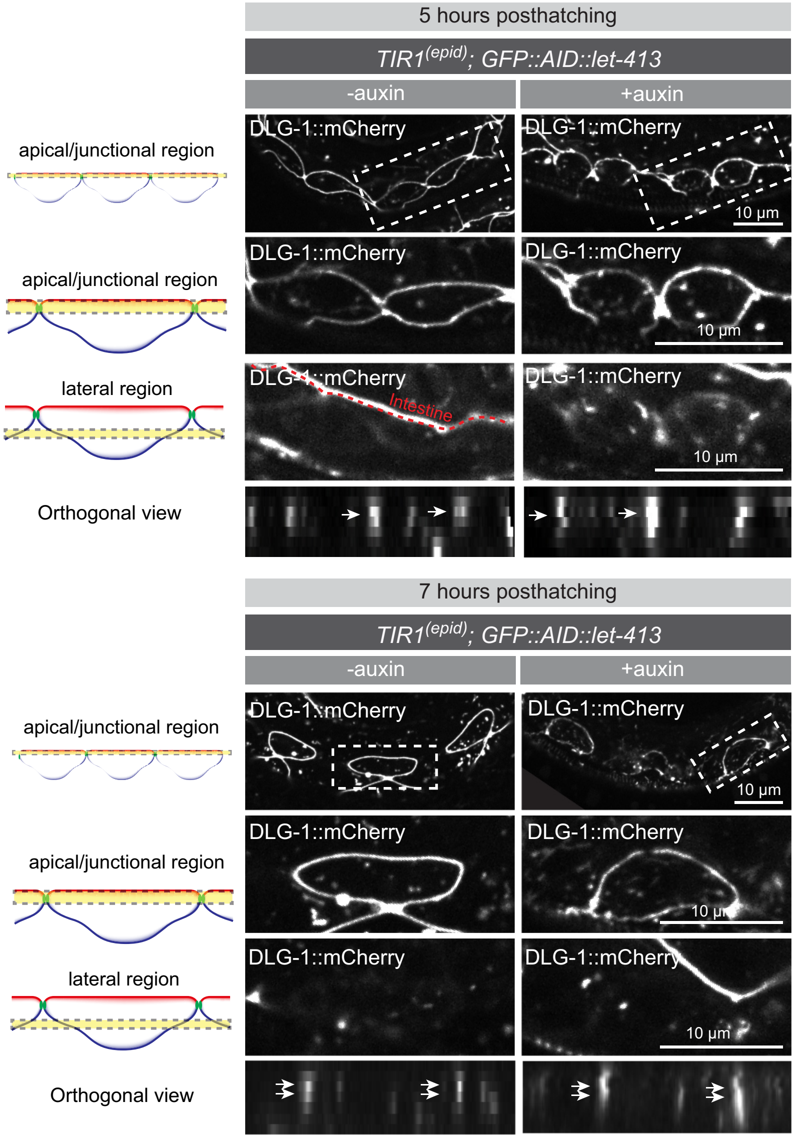

S4 Fig. LET-413 depletion disrupts the localization of DLG-1. Distribution of DLG-1::mCherry in the epidermis of *Pwrt-2::TIR1::BFP; GFP::AID::let-413; dlg-1::mCherry* animals without (-auxin) and in the presence of auxin (+auxin) at 5 h and 7 h post hatching (strain BOX527). Boxed region in top overview panels is shown enlarged below. Related to Fig 5. Note that 7 h timepoint is also shown in Fig 5E, and replicated here for ease of comparison between time points.
