# Supplementary material for: Caenorhabditis elegans LET-413 Scribble is essential in the epidermis for growth, viability, and directional outgrowth of epithelial seam cells
Source: PLoS Genet. 2021 Oct 21;17(10):e1009856. doi: 10.1371/journal.pgen.1009856 (PMC8570498; doi:10.1371/journal.pgen.1009856)
Supplement: S5 Fig — (A) Distribution of DLG-1::mCherry upon depletion and degradation of HMR-1 at 10 h post hatching. Left panels are controls not expressing CRE and not treated with auxin, right panels show animals expressing CRE and treated with auxin. Genotypes are hmr-1::LoxP::AID::GFP::loxP; Pwrt2::GFP::PH Pwrt-2::GFP::H2B for the control (strain BOX832) and hmr-1::LoxP::AID::GFP::loxP; Pscm::CRE; Pwrt-2::TIR1::BFP; Pwrt2::GFP::PH Pwrt-2::GFP::H2B for the CRE and auxin-treated animals (strain BOX832). (B) Quantification of HMR-1 intensity across the hyp7–seam junction in animals depleted of HMR-1 as in A (+ auxin), and in control animals (- auxin). Graph shows mean mCherry fluorescence intensity ± 95% CI. N = 5 animals for control and 5 animals for the HMR-1-depleted conditions. (C, F) Distribution of HMR-1::GFP upon depletion of DLG-1 at 4 h (C) and 10 h (F) post hatching. Left panels are controls not treated with auxin, and right panels are animals in which DLG-1 was depleted by auxin treatment. Genotype is hmr-1::GFP; Pwrt-2::TIR1::BFP; dlg-1::mIAA7::mCherry (strain BOX825). (D, E, H, I) Quantification of DLG-1::mIAA7::mScarlet or HMR-1::GFP intensity across the hyp7–seam junction in animals depleted of DLG-1 as in C, F (+ auxin), and in control animals (- auxin). Graph shows mean mCherry fluorescence intensity ± 95% CI. N = 6 animals for control and 5 animals for the DLG-1-depleted conditions. Related to Fig 6. (PDF) [file pgen.1009856.s005.pdf]

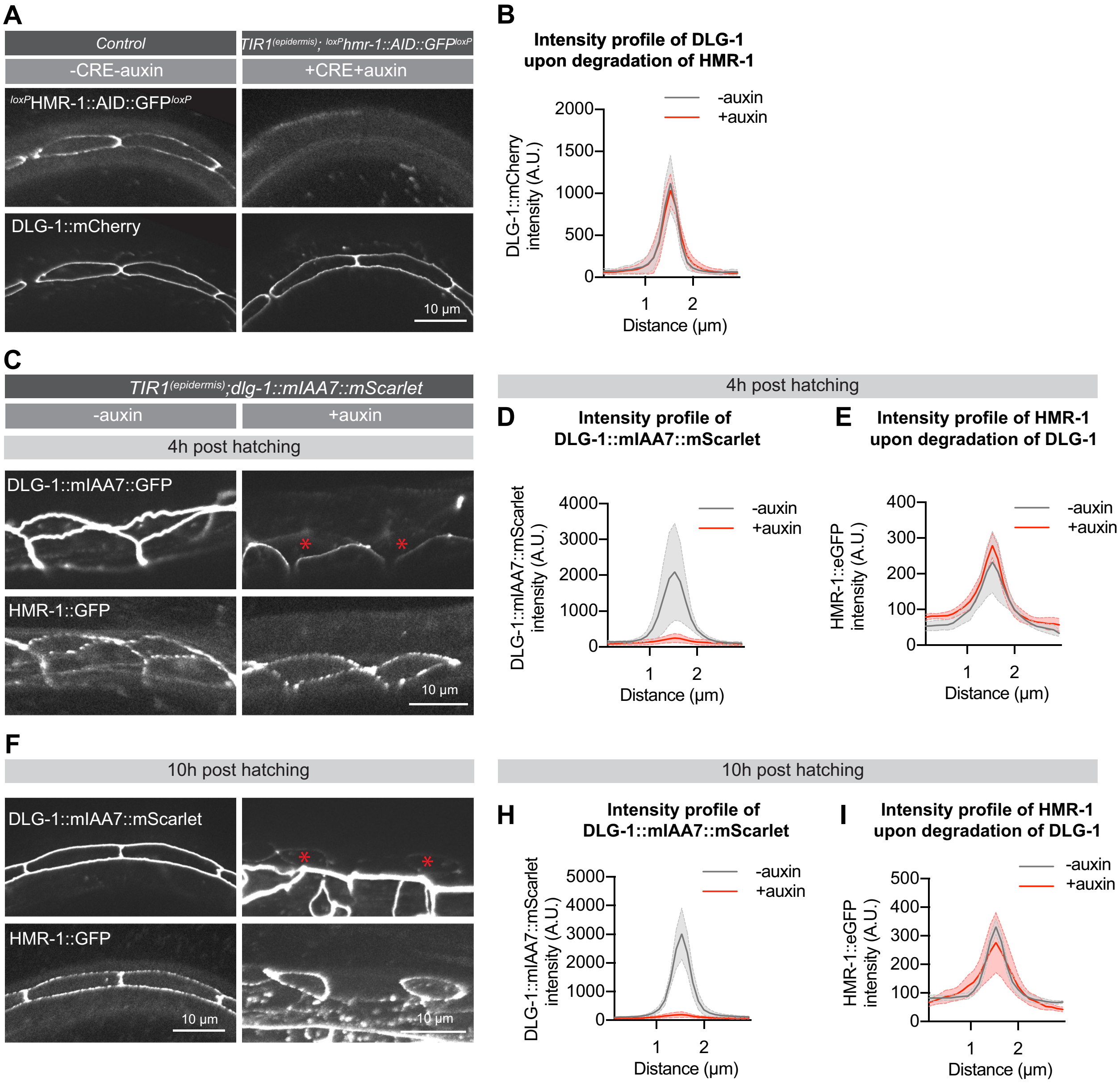

S5 Fig. DLG-1 and HRM-1 localize independently in the epidermis. (**A**) Distribution of DLG-1::mCherry upon depletion and degradation of HMR-1 at 10 h post hatching. Left panels are controls not expressing CRE and not treated with auxin, right panels show animals expressing CRE and treated with auxin. Genotypes are *hmr-1::Lox-P::AID::GFP::loxP; Pwrt2::GFP::PH Pwrt-2::GFP::H2B* for the control (strain BOX832) and *hmr-1::LoxP::AID::GFP::loxP; Pscm::CRE; Pwrt-2::TIR1::BFP; Pwrt2::GFP::PH Pwrt-2::GFP::H2B* for the CRE and auxin treated animals (strain BOX832). (**B**) Quantification of HMR-1 intensity across the hyp7–seam junction in animals depleted of HMR-1 as in A (+ auxin), and in control animals (-auxin). Graph shows mean mCherry fluorescence intensity ± 95% CI. N = 5 animals for control and 5 animals for the HMR-1-depleted conditions. (**C, F**) Distribution of HMR-1::GFP upon depletion of DLG-1 at 4 h (C) and 10 h (F) post hatching. Left panels are controls not treated with auxin, and right panels are animals in which DLG-1 was depleted by auxin treatment. Genotype is *hmr-1::GFP; Pwrt-2::TIR1::BFP; dlg-1::mIAA7::mCherry* (strain BOX825). (**D, E, H, I**) Quantification of DLG-1::mIAA7::mScarlet or HMR-1::GFP intensity across the hyp7–seam junction in animals depleted of DLG-1 as in C, F (+ auxin), and in control animals (- auxin). Graph shows mean mCherry fluorescence intensity ± 95% CI. N = 6 animals for control and 5 animals for the DLG-1-depleted conditions. Related to Fig 6.
