# Supplementary material for: Caenorhabditis elegans LET-413 Scribble is essential in the epidermis for growth, viability, and directional outgrowth of epithelial seam cells
Source: PLoS Genet. 2021 Oct 21;17(10):e1009856. doi: 10.1371/journal.pgen.1009856 (PMC8570498; doi:10.1371/journal.pgen.1009856)
Supplement: Attachment — Submitted filename: Response to the reviewers.docx [file pgen.1009856.s010.docx]

We thank the reviewers for their critical reading of the manuscript and their insightful comments. In response to their suggestions, we have made two major changes to the manuscript. First, we have de-emphasized the potential role of LET-413 acting upstream of Rac or Cdc42. We agree with the reviewers that our original data do not support this conclusion strongly enough. Additional experiments in this direction were still inconclusive. While mammalian data still make regulation of actin dynamic an attractive LET-413 function, our data on the role of DLG-1 in seam cell extension are much more solid. We have moved the actin section to earlier in the manuscript, shortened it, and no longer make the conclusion that LET-413 may act upstream of small GTPases.

The second change is the expansion of our analysis of the role of the two junctional complexes (DLG-1/AJM-1 and cadherin-catenin) in seam cell extension. We show that HMR-1 E-cadherin is not required for this process. We also show that HMR-1 and DLG-1 have distinct localization patterns, and that these two proteins do not depend on each other for their localization. The role of LET-413 in seam cell extensions therefore seems to be specifically mediated (largely) by DLG-1.

We hope that the reviewers agree that these changes strengthen the manuscript. A point-by-point response is below.

# Reviewer 1

The conserved protein Scribble is a scaffolding molecule that contributes to cell polarity, cell migration and tissue structure across animals. The C. elegans Scribble homolog let-413 is essential for embryonic morphogenesis. Since let-413 mutants die during embryogenesis, post-embryonic roles of let-413 have been unknown. Here, Boxem and colleagues describe phenotypes induced by depleting LET-413 post-embryonically via the auxin-inducible degron system. Interestingly, no clear defects were observed upon depletion of LET-413 in intestinal cells; however, LET-413 depletion produced defects in epidermal seam cells, which ultimately led to larval arrest and lethality.

This is a straightforward and interesting study that describes novel (to my knowledge) roles of LET-413 in the larval epidermis. The manuscript is well written and the data are, for the most part, clearly presented. I have some comments for the authors to consider, but no serious concerns that would preclude publication.

*I found the images and quantification in the first half of Figure 4 somewhat unconvincing.*

We have changed the representation of the figures to make the representation clearer. Due to the low expression levels of PAR-6 and LGL-1, however, there are unfortunately limits to the clarity of the images we can obtain. We address the quantification below.

*In Figure 4A, are we seeing a lateral or apical focal plane?*

We have added schematics of the focal plane shown in the images to the figure to clarify this.

*In Figure 4B, I’m not sure a line scan is the best form of quantification. There is clearly still cortical PAR-6, but without knowing what focal plane we’re in, I don’t know how to interpret this.*

We have now indicated the areas shown in the images in a schematic and clarified in the legend and methods that quantifications are done at the apical/junctional plane. We have also tried the quantification in a different way, tracing a line around the perimeter of the cells and quantifying the total fluorescence. The results are the same as using the original method. In order not to change approach for this one experiment, we have kept the original quantification. The results of the alternative method are shown here, and we would be open to adding this as a supplemental figure.

*I like that Figure 4C shows both apical and lateral focal planes, but it’s hard to see what is going on because the signal:background is poor. While I generally applaud the use of endogenous tags for everything, this might be a rare case where a seam cell-specific GFP::PAR-6 transgene would give clearer results.*

We have tried analyzing a different, transgenic, line. We imaged strain FT17 (Ppar-6::par-6::GFP::par-6utr generated by bombardment), which has a higher overall expression. However, the transgene had a worse signal-to-background ratio than the endogenous tag and was more difficult to quantify. We therefore left the original data in place, and hope that the other improvements to this figure make our case more clearly.

*For Figures 4D-E, were both apical and lateral planes acquired? If not, how do we know that LGL-1::GFP is not mislocalized apically?* *My suggestion would be to acquire closely-spaced Z stacks of these animals, and then show reconstructed YZ images (corresponding to a transverse section across the body of the animal) to clearly visualize both the apical and lateral membranes. Quantification could then directly compare apical vs. lateral fluorescence intensity.*

For all our imaging we routinely capture complete Z stacks at 0.25 um spacing. We have added the suggested orthogonal (YZ) views. The seam cells are not particularly nice looking in orthogonal views due to their irregular shape and other tissues abutting the seam cells on the basal side. Particularly the intestine, which has bright expression of polarity and junction proteins, is confounding. Thus, while the orthogonal images are not suitable for quantification, they do help show that the localization in these figure panels is unchanged upon LET-413 depletion.

*It’s difficult to see what’s going on in Figure 3E. Why is the P cell brightly labeled by GFP::PH in the -auxin but not the +auxin condition? If this reflects a trivial issue like choice of focal plane, I suggest the authors acquire new images that show the phenotype clearly.*

This is not a trivial issue but appears to be an aspect of the phenotype. We have not investigated this further, but indeed the P cells in +auxin condition lose expression of GFP::PH. In the text we we commented on this: “..., as we noticed that LET-413 depletion results in reduced expression in P cells of the Pwrt-2-driven GFP::H2B and GFP::PH marker proteins (Fig. 3E)”. We again refer to it in the discussion.

*The authors could do a better job making the experimental conditions clear from their figure legends. For every figure panel, the figure panel should include 1) What strain is being imaged – ideally including the strain name so that the reader can cross-reference to the strain table; 2) What larval stage is being imaged; and 3) How many replicates the image represents. This information is present in some figure legends, but it’s not consistent throughout the paper.*

We have added the strain names to the legends and double-checked to make sure that N numbers are present for all quantifications and images representing a partially penetrant phenotype. We also included the time of development. In most case we cannot assign an exact larval stage, as LET-413 depleted animals do not develop normally. Instead, we indicate hours post hatching.

# Reviewer 2

Riga et al., show that inducible degradation can be used to overcome the need of Scrib (LET-413) during C.elegans embryogenesis to study tissue-specific functions during late larval development. This led to the identification of a new role for LET-413 in the polarized outgrowth of epidermal seam cells. The phenotype is very well characterized by live imaging to follow seam cell division pattern and the subsequent steps until showing the specific inability of seam cells to form protrusions. This finding is very interesting by providing the first evidence that Scrib plays a role in protrusive cell shape change in invertebrates, which can bring new parallels with the roles in cell migration described in vertebrates. The paper is generally well-written and given the broad relevance of the Scrib protein, it could gather general interest by cell and developmental biologists.

The manuscript also shows that another component of the Scrib module, Dlg, has a similar role in the regulation of protrusive activity and proposes that not only Dlg but also CDC42 and the CED-10 RAC act downstream of LET-413. The conclusions taken in this second part of the manuscript are not completely supported by the data. For instance, the manuscript provides evidences against a fundamental role of CDC42/RAC by showing that their inactivation does not affect the protrusive activity of seam cells. Moreover, compelling experiments linking the role of GTPases to LET-413 function are absent. Addressing this and some other specific points described below should be considered before publication.

## Major issues

*1) line 256 “ We also did not detect apical invasion or reduced basolateral levels of LGL-1 (Fig. 4D, E).” Legend states that this figure is a maximum projection of apical and junctional domain and so evidence of the maintenance of basolateral levels of LGL-1 should be provided by imaging planes similar to the ones in Fig. 4C for PAR6. Moreover, there is an increase in the peak of Lgl intensity after auxin treatment in the graph of Fig. 4E, which is intriguing and should be analyzed statistically to clarify if there is apical invasion.*

We have changed the display of the figure to show the apical domain, a mid-plane, and an orthogonal view. None of the views support apical invasion. We notice that the levels at the cortex indeed seem somewhat elevated. Statistical analysis indicates that this difference is significant (with a modest p value of 0,02 using a t-test to compare peak levels). We hesitate to conclude that the levels are indeed altered. The cells are smaller than in controls, which may account for the minor increase in levels, and the expression is somewhat variable in depleted animals. Coupled with the lack of LGL-1 signal at the apical domain, we don’t feel our data supports that LGL-1 localization changes upon LET-413 depletion.

*2) The manuscript shows clearly that LET-413 function is both required for the organization of cadherin-based junctions and to maintain the normal localization pattern of Dlg at the junctional level (Fig. 4F-4G). This result helps defining the functional hierarchy of the Scrib module components. However, the finding that Dlg mislocalization only becomes apparent few hours after junction disruption brings in the alternative hypothesis that defects in junctional organization are the underlying cause of Dlg mislocalization. The authors should tackle experimentally this alternative or at least discuss it.*

We agree with this point and have greatly expanded our investigation of the role of the different junctional complexes in seam cell extension. We have added an investigation of the contribution of the cadherin-catenin complex to the phenotype and examined the effects of DLG-1 loss on HMR-1 E-cadherin and vice versa. We find that loss of HMR-1 does not result in defects in the localization of DLG-1 and does not cause a loss of seam cell extension in L1 larvae. Vice versa, DLG-1 loss did not result in a concomitant loss of HMR-1. Finally, HMR-1 and DLG-1 have distinct localizations during seam cell extension: while DLG-1 is located in a ring around the base of the extensions, HMR-1 is present in the extensions themselves. Taken together, the DLG-1/AJM-1 and cadherin/catenin complexes appear to have distinct functions in seam cell extension, and the seam cell extension defects of LET-413 animals appear to be largely mediated by the DLG-1/AJM-1 complex.

*3) The manuscript suggests that DLG-1 acts downstream of LET-413 while showing a weaker phenotype of DLG-1 induced degradation. The evidence for the less dramatic effect is robust, but the phenotype should be further analyzed to clarify the relation with the phenotypes presented by LET-413 depletion. In particular, it would be worth to analyse HMR-1 localization to evaluate if it was equally affected by DLG-1 depletion. Considering if different effects in cadherin-based junctions could be related to the additional features of the LET-413 phenotype is particularly relevant due to the connection between cadherin-based junctions and the cytoskeleton.*

We have now analyzed the localization of HMR-1 in a DLG-1 depleted background, as well as the effect of HMR-1 depletion on seam cell extension. The loss of DLG-1 does not appear to affect the localization of HMR-1, and the depletion of HMR-1 does not cause defects in seam cell extension. Thus, the major contributor to seam cell extension appears to be DLG-1.

*4) Figure 5G showing a basolateral expansion phenotype that is specific of Dlg is not completely clear. This phenotype should be quantified or at least better represented to clarify if the authors refer to basolateral expansion in relation to control (basal seems larger in control) or if there are also differences to LET-413 depletion. In addition, it could be better reasoned how and if this phenotype is linked to the idea that LET-413 has additional activities independently of Dlg.*

We agree that our analysis of this phenotype was not thorough enough. We have revisited the phenotype of DLG-1 depletion. In some animals, we observed the larger basolateral domain originally described, but with higher N numbers this phenotype was not very penetrant. More commonly, we observed membrane-enveloped blebs appearing in the seam cells, which are never observed in LET-413 depleted animals. We have added a video of this phenotype. We have adjusted our description of the difference between DLG-1 and LET-413 depletion, and no longer make a strong conclusion about basolateral expansion.

*5) Since the additional activities of LET-413 could be simply explained by the weaker efficiency of depletion, the authors should provide stronger arguments to support the conclusion that there are activities independent of DLG-1. Evidence such as “LET-413 depletion does not result in a complete loss of DLG-1 yet still causes a more complete block of seam cell outgrowth “ is not sufficient because it is possible that localized Dlg is not functional in this specific process without available LET-413, such that the stronger depletion of LET-413 would create stronger functional disruption of DLG-1.*

We have done our best to improve the depletion of DLG-1. We tried using a different degron sequence, which dramatically improved DLG-1 depletion in the intestine. We are preparing a manuscript on this new degron sequence. In the seam cells the improvement in DLG-1 depletion was more modest, and we were still unable to deplete all DLG-1 (leaving around 6% remaining). We have rephrased any discussions to clarify that remaining DLG-1 levels may account for the remaining membrane activity we observe.

*6) In the final part of the manuscript the authors test the role of Rho family GTPases in seam cell extension to investigate if these proteins could be important in the promotion of cell protrusion controlled by LET-413. Overexpression of active forms of Rac and CDC42 are well known to promote cytoskeleton reorganization and so inducing their ectopic activation would be anticipated to disrupt cytoskeleton organization regardless of a relevant function in normal conditions. Thus, ectopic overexpression experiments are insufficient to conclude about a fundamental role in regulating protrusion formation at the leading edge of extending seam cells. In fact, RNAi and expression of dominant-negative versions provide evidence that neither inactivation of Rac nor CDC42 produce any relevant defect (Fig. 6C and 6E). Although this is discussed as resulting of possible redundancy, the manuscript still lacks data to evaluate redundancy or the efficiency of inactivation. Moreover, there is no data supporting a genetic or molecular link between LET-413 and activity of these GTPases. The authors may consider testing for phenotypic interaction (e.g. co- depletion Dlg and CDC42/Rac inactivation could bring the function of CDC42/Rac as the additional activities that are responsible for the weakened phenotype of Dlg).*

It has proven very difficult to address the roles of actin regulators in seam cell extension. We have performed additional double dominant negative experiments but were not able to block the seam cell extensions. We were able to obtain a partially penetrant block by combining CED-10 dominant negative with triple Rac inactivation. One major difficulty is that if actin is disrupted too severely, the seam cells will not divide and arrest before the stage of seam cell extension. While it seems likely that there is a direct or indirect link to actin, we have toned down the conclusions as suggested below.

*Given the current data, the manuscript should be considerably revised to tone down any conclusions related to the function of GTPases and its relation with the identified role for LET-413. For instance, the available data is not compatible with “an important role for the CED-10 Rac and CDC-42 GTPase proteins in reorganizing the actin cytoskeleton and driving anterior–posterior directed outgrowth of the seam cells. “; “Finally, we demonstrate that the Rho-family GTPases CED-10 Rac and CDC-42 can regulate seam cell outgrowth and may also function downstream of LET-413”; “Our data build upon these observations and support an important role for the regulation of actin dynamics by CED-10 Rac and CDC-42 in seam cell extension.”*

We appreciate the comment. We have changed the order of the data in the paper to shift the focus from actin to the role of DLG-1, where our data is more solid.

*7) The authors state in the abstract (lines 25 to 27, similar sentence in introduction in lines 88 to 90 ): “We show that the role of LET-413 in seam cell outgrowth is mediated at least in part by the junctional component DLG-1 discs large, which appears to restrict protrusive activity to the apical domain.” These sentences could be misleading as the word “restrict” suggests that DLG-1 blocks protrusive activityin all other regions of the membrane to ensure that it is limited to the apical domain. If my understanding is correct, the manuscript did not show sufficient evidence of the presence ectopic protrusive activity in the basolateral domain upon DLG-1 depletion to justify this conclusion.*

We have now better analyzed the role of DLG-1 and HMR-1 in seam cell extensions, which has changed the discussion. We have no longer made this particular point, as we agree with the reviewer that our data does not support this strongly enough.

## Minor issues

*1) Related to Major issue 1: the authors could reinforce the idea that loss of LET-413 does not disrupt apicobasal polarization by showing that junctions were not mispositioned along the apical-basal axis with imaging for HMR-1 across the lateral plane such as done for PAR6:GFP in Fig. 4C.*

We now present additional focal planes and orthogonal views for HMR-1 and DLG-1.

*2) Discussion line 492: Although the authors did a great work at discussing the phenotypic difference between seam-specific RNAi from a previous study and induced protein depletion, it may be also relevant to discuss whether these differences could be related to the distinct timing of effective protein depletion.*

This could certainly be the case. Unfortunately, in the Brabin et al. paper, the *let-413* and *dlg-1* data are listed as not shown, and for the *ajm-1* data the stage of the worm shown is not represented. Without that information we cannot speculate on whether the RNAi would take effect sooner or later than our degradation. We therefore added only a short statement that “Alternatively, the observed difference could be due to differences in the timing of protein depletion by RNAi and auxin-inducible degradation.”

*3) Discussion line 528-531: It may be worth to consider that the distinct hierarchy defined in these cases could be related to the specific subcellular location that is being accessed in each Drosophila tissue. The Scrib module is purely located at the junctional level (septate junction) in the Drosophila midgut (Chen et al., Plos Biol) where Scrib is upstream of Dlg. In contrast, the studies in the follicular epithelium (Khoury and Bilder, PNAS, 2020; Ventura et al., Development, 2020) analyzed the basolateral localization of Scrib and show that this is indeed affected in dlg mutants. I should also note that earlier studies in a different Drosophila tissue, embryonic epithelium (Bilder et al, Science 2000), also reported Dlg upstream of Scrib.*

We thank the reviewer for this detailed information. We have expanded the discussion to include the data by Bilder et al in the embryonic epithelium (co-dependency), point out the differences in localization, and altered the point of the discussion slightly to indicate that the relationship between these proteins has not been investigated in all *C. elegans* tissues in which they are expressed.

# Reviewer 3

## Summary

This manuscript from the Boxem group extends their work on tissue-specific proteomics using degron technology to examine the role of a key, conserved polarity protein, LET-413/Scrib and its interactors, including DLG-1/Discs large, in postembryonic differentiation and morphogenesis in the embryonic epidermis (hypodermis) in C. elegans.

The role of LET-413 and DLG-1 in apicobasal polarity and junctional integrity was established long ago by papers from the Labouesse, Bossinger, and Hardin groups. However, while a bit of structure-function on LET-413 was subsequently performed, little has been done to examine the role of this polarity cassette in postembryonic development, which is the subject of this paper. As such this paper could be very appropriate for PLoS Genetics and should be of interest to its readers.

## Interesting findings

- A basic finding of this work is perhaps not surprising: LET-413 is required in the epidermal epithelium for growth, viability, and junctional stability/maintenance. What is more surprisingly (but consistent with previous reports) is that LET-413 is not essential in the larval intestine.
- The additional, novel finding here is that LET-413 and DLG-1 play a role in the extension of seam cells (lateral epidermal cells). These cells are interesting for several reasons: they act as a stem population, show polarized division patterns, and they undergo interesting patterns of segregation from the surrounding epidermal syncytium.
- Epidermal depletion of LET-413 caused severe defects in the localization pattern of both HMR-1 and DLG-1. HMR-1 puncta became sparser, while DLG-1 was no longer localized in a continuous belt and instead localized to discontinuous short stretches.
- The authors suggest that the DLG-1/AJM-1 junctional complex is involved in spatially restricting the protrusive activity to the apical domain. This is an interesting idea, since this would be the first such evidence for such a role for DLG-1 that I am aware of. The effects of DLG-1 depletion suggest that there is an effect on basolateral localization of seam cells extensions.

## Conclusions

While the basic AID methodology and associated results are interesting, there are several questions that need to be resolved regarding how LET-413 and DLG-1 act in seam cell before this work is suitable for PLoS Genetics.

## Major comments

*Based on analysis of constructs designed to perturb Rho family GTPase signaling, the authors suggest that the extension process is a migratory event in which LET-413/DLG-1 restricts "protrusions" to the apical domain. This is an interesting and reasonable idea, if the actin-containing extensions are in fact protrusive. However, it seems unclear if this is the case. The authors state that "In control animals, the seam cells showed signs of active membrane dynamics immediately following cell division, displaying small filopodia-like protrusions around the cell body. The apical domains of the seam cells then formed larger lamellipodium-like protrusive fronts directed towards the adjacent seam cells", whereas these seem to disappear in the let-413-depleted larvae. Showing that these "protrusive fronts" really are protrusive, rather than relatively quiescent subapical cortex, seems important for the interpretation of the entire story here. Can the authors perform some live imaging to ascertain this?*

We have done the suggested live imaging of extending seam cells (Videos S1 and S2). The cortex is quite active, indicating that these are active processes and not a passive expansion.

*This issue is exacerbated by the refractory nature of these "protrusions", as the authors state: "We were not able to block formation of seam cell protrusions through mutation, RNAi, or expression of dominant negative variants of Rac family members and cdc-42." While I am agnostic on whether these are indeed "protrusions", a skeptic might say this is strong presumptive evidence that they are not. Making a bit of headway on the function of these extensions seems important. Most would worry about the CA expression results here is there are not complementary effects using loss-of-function or dominant negatives.*

We have expanded our approaches to inactivate Rac and Cdc42. While we are able to observe disconnected seam cells, the effect is never as penetrant or severe as upon depletion of LET-413. In addition, we have expanded our analysis of the role of the junctional complexes. These data have changed the emphasis of the manuscript, and we hope that we now provide a more balanced discussion.

*Why did the authors not use ZF1 or AID technology to deplete Rho family GTPases, given that this approach is used so much in the paper? Did that also lead to no phenotype?*

We decided against this approach due to the large number of Rho family GTPases that would need to be tagged (3 Racs, Rho, and Cdc42), and the worry that tagging these small proteins would affect their functioning. It would also be very difficult to time their depletion correctly, hence it would still not be a very clean experiment. In the end we decided that the time investment in generating these tools would likely not pay off.

*Since LET-413 blocks all protrusion formation it is hard to know if the main activity of LET-413 is as an upstream regulator of DLG-1 or instead it acts through other mechanisms. The authors admit this, yet suggest that LET-413 might be upstream of CDC-42: "these results are consistent with LET-413 acting upstream to regulate CDC-42 activity, this cannot be concluded from these data as no protrusions are formed when LET-413 is depleted." I think this is correct. Making additional headway on this points would improve this paper, but seems less important than strengthening the story related to protrusions.*

We have focused on strengthening our data on the junctional complexes and on demonstrating that protrusion formation is an active process.

## *Other comments/questions*

*I wish to strongly commend the authors for their honest interpretations and caveats throughout much of this paper. They were a pleasure to read.*

*The P cell phenotype is interesting. Can the authors say anything more about this phenotype?*

We think so too, but we have not done any further investigation into the cause of the phenotype, and can’t really speculate on it without any data.

*What is interesting here is that the apicobasal position of the puncta is maintained, which is different from the effects of LET-413 deletion in some other tissues (e.g., embryonic epidermis and intestine). Do the authors have a suggestion as to the biological difference? Is it the presence of the cuticle?*

This is a good point. There does not seem to be any a priori reason why basolateral expansion is not possible in the seam cells. Nevertheless, the different tissues do rely to different extents on polarity regulators in larvae, and in these established cells other mechanisms may act to keep PAR-6 apical and LGL-1 basal.
